# Novel scoring tool of hypoxemic respiratory failure and pulmonary hypertension for defining severity of persistent pulmonary hypertension of newborn

**DOI:** 10.1038/s41372-023-01762-w

**Published:** 2023-08-25

**Authors:** Sage Timberline, Avni Bhatt, Sherzana Sunderji, Daniel J. Tancredi, Satyan Lakshminrusimha, Heather Siefkes

**Affiliations:** 1 Department of Pediatrics, University of Virginia, Charlottesville, VA USA; 2 School of Medicine, University of California, Davis, Sacramento, CA USA; 3 Department of Pediatrics, University of California, Davis, Sacramento, CA USA

**Keywords:** Outcomes research, Risk factors

## Abstract

**Objective:**

To obtain preliminary validity data for a hypoxemic respiratory failure/pulmonary hypertension (HRF/PH) score for classifying persistent pulmonary hypertension of the newborn (PPHN).

**Study design:**

Retrospective chart review of 100 consecutive neonates admitted to a Children’s hospital from 2016–2021 with PPHN, gestational age ≥34 weeks, and echocardiograms in the first week. We assessed the correlation between HRF/PH score and short-term outcomes using linear and logistic regressions.

**Results:**

HRF/PH scores ranged 2–12 (mean 8.5), and were classified mild (0–5), moderate (6–10), and severe (11–15), with 6%, 77% and 17% infants in respective categories. HRF/PH score category correlated with invasive ventilation, nitric oxide, high frequency ventilation, vasoactive infusions, extracorporeal life support and death. HRF/PH score category did not correlate with duration of support or length of stay.

**Conclusion:**

The HRF/PH score offers a promising representation of disease severity for PPHN. The tool requires further validation in prospective studies and evaluation for long-term outcomes.

## Introduction

Failure to transition from the fetal to the neonatal period [1, 2] with a reduction in pulmonary vascular resistance (PVR) can lead to hypoxemic respiratory failure (HRF) and persistent pulmonary hypertension of the newborn (PPHN) [3]. HRF and PPHN may be conservatively managed with lung recruitment (with ventilation and surfactant if indicated), oxygen supplementation, inhaled nitric oxide (iNO), and in some cases extracorporeal life support (ECLS). Randomized controlled trials (RCTs) seeking to optimize treatments such as iNO [4–8], milrinone [9], and surfactant [10] have traditionally used oxygenation index (OI) as an inclusion criterion. Occasionally, some of these trials have added the presence of PPHN by echocardiography as a dichotomous variable and an additional criterion for inclusion [4]. The severity of hypoxemia and pulmonary hypertension contribute to the pathophysiology of HRF and PPHN [11]. However, to our knowledge, there are no RCTs that have assessed the severity of HRF *and* pulmonary hypertension as combined inclusion criteria or as the primary outcome. Most of these trials have evaluated death or cannulation for ECLS as the primary outcome. Given the low frequency of death/ECLS in PPHN (with the exception of congenital diaphragmatic hernia – CDH), studies that are planned with this outcome require a large sample size. Development and validation of a score that combines assessment of the severity of hypoxemia [12] and pulmonary hypertension [13] may offer alternate criteria for inclusion and/or primary outcome in trials evaluating HRF/PH [14].

In a collaborative effort with neonatologists, pediatric cardiologists, and pediatric intensivists at our institution, we developed an HRF/PH score that incorporates a level of hypoxemia and echocardiographic findings of PH. In this study, we then aimed to evaluate this score in a retrospective review of infants with PPHN and how well it correlated with short-term outcomes such as ECLS/death, length of stay (LOS), and need/duration of inpatient therapies.

## Methods

This study received IRB exemption status (ID: 1668854-3).

### Study population

A retrospective chart review was performed of newborn infants (0–7 days of postnatal age) admitted to a level IV academic neonatal intensive care unit (NICU) from January 2016 to December 2021. Inclusion criteria included a documented diagnosis of PPHN by ICD 9 or 10 codes, gestational age ≥34 weeks, and echocardiogram in the first 7 days after birth. The range of 2016–2021 was chosen based on annual estimates to target 100 total patients to fit the scope and feasibility of a retrospective study geared towards obtaining preliminary score validity data. Those with CDH, Trisomy 21, and hypoxic ischemic encephalopathy (HIE) undergoing therapeutic hypothermia were included. Exclusion criteria included congenital heart disease other than atrial septal defect/patent foreman ovale (ASD/PFO), patent ductus arteriosus (PDA), or ventricular septal defect (VSD) < 2 mm.

### Outcomes

The primary outcome was death/ECLS before discharge. Other outcomes were the length of stay (LOS), survival to discharge, and the use and duration of supportive care required through the infant’s hospital course, including IMV, high frequency oscillatory ventilation (HFOV), iNO, need for vasoactive infusions and maximum vasoactive-isotropic score (VIS – equation in supplement).

### Hypoxic respiratory failure/pulmonary hypertension score

**Fig. 1:**
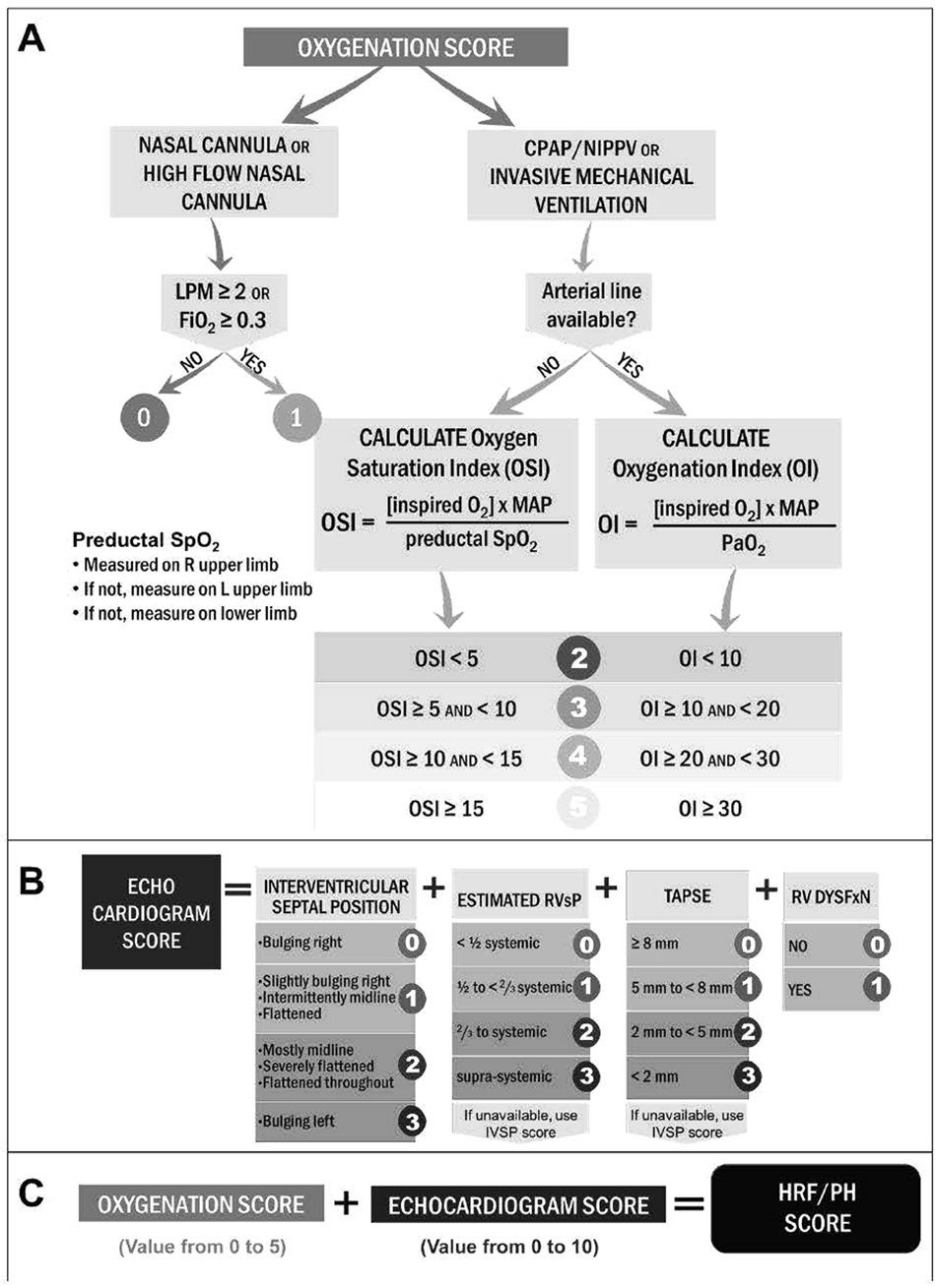
Components of hypoxic respiratory failure/pulmonary hypertension (HRF/PH) score. **A** Oxygenation score or hypoxemic respiratory failure (HRF) representing the level of oxygen support at the time of the first echocardiogram. **B** Echocardiogram score (PH) representing the severity of pulmonary hypertension based on first echocardiogram findings. **C** Oxygenation score (HRF) and echocardiogram score (PH) were added to form a final HRF/PH score. LPM liters per minute, FiO_2_ fraction of inspired oxygen, CPAP continuous positive airway pressure, NIPPV non-invasive positive pressure, OSI oxygen saturation index, OI oxygenation index, MAP mean airway pressure, SpO_2_ oxygen saturation, PaO_2_ arterial partial pressure of oxygen, IVS interventricular septum, RVsP estimated right ventricular systolic pressure, TAPSE tricuspid annular plane systolic excursion, RV dysfxn right ventricular dysfunction. *Copyright Avni Bhatt*.

Prenatal, perinatal, and postnatal demographic data were collected to assess potential risk factors that contributed to PPHN for each newborn infant. Data related to the earliest complete transthoracic echocardiogram for each infant were reviewed to develop the HRF/PH score, which was divided into an oxygenation (HRF) score and an echocardiogram (PH) score (Fig. 1). The oxygenation score was assigned to each neonate based on the respiratory support and oxygenation at the time of the echocardiogram. Those receiving either nasal cannula or high flow nasal cannula (HFNC) scored 0 or 1, with a score of 1 indicating a flow rate ≥2 liters per minute, or a fraction of inspired oxygen (FiO_2_) at 0.3 or greater. Those who required higher levels of oxygenation support with continuous positive airway pressure (CPAP), nasal intermittent (non-invasive) positive pressure ventilation (NIPPV), or invasive mechanical ventilation (IMV) received a score of 2 or higher. If an arterial gas was available within four hours of the echocardiogram, the OI (FiO_2_*100*Mean airway pressure/PaO_2_) was calculated. The use of oxygen saturation by pulse oximetry (SpO_2_) instead of arterial partial pressure of oxygen (PaO_2_), reduces the need for an indwelling arterial line and enables assessment of preductal and postductal oxygenation in a continuous and non-invasive manner. Hence, among infants without an arterial line, the oxygen saturation index (OSI = FiO_2_*100*Mean airway pressure/SpO_2_) was calculated using preductal SpO_2_, which was preferentially measured on the right upper limb > left upper limb > lower limb. Increasing values of OI or OSI in these patients corresponded to a higher oxygenation score, with an OSI < 5 or OI < 10 indicating an oxygenation score of 2 and increasing incrementally (Fig. 1A).

The PH component of the HRF/PH score analyzed the first complete echocardiogram for the interventricular septum (IVS) position, tricuspid annular plane systolic excursion (TAPSE), estimated right ventricular systolic pressure (RVsP), and presence of right ventricular dysfunction. If the echocardiogram report available in the chart did not contain all of these components, the echocardiogram was reviewed by a single pediatric cardiologist (SS) on the research team to provide the missing data and validate measurements. IVS position has been correlated to the degree of right ventricular pressures [15]. Specifically, with increasing right ventricular pressures, the short axis geometry of the left ventricle is distorted and leads to progressive flattening of the IVS during systole. As such, the IVS position was scored from 0–3 based on its position in the parasternal short axis. TAPSE, which was measured from a standard apical 4 chamber window and graded from 0–3 based on the numerical measurement, quantifies the systolic excursion of the tricuspid annulus in the longitudinal direction of the right ventricular free wall and represents a right ventricular longitudinal function. The RVsP was calculated and graded from 0–3 based on the peak tricuspid regurgitation jet velocity and the use of the simplified Bernoulli equation: RVsP = 4(v)^2^, where v is the peak velocity of the tricuspid valve regurgitant jet measured in meters/second. The tricuspid jet was determined either in the parasternal short axis or the apical 4 chamber - whichever image provided the most complete Doppler waveform. If the RVsP was unable to be determined by the tricuspid regurgitant jet due to the lack of valve insufficiency, the IVS position score was multiplied by 2. Right ventricular dysfunction was assigned a score of either present or absent based on the interpretation and experience of the imaging-trained pediatric cardiologist assessing all echocardiograms for the study. PH score was then assigned to each newborn from these components (Fig. 1B). The oxygenation (HRF) and echocardiogram (PH) scores were totaled for a cumulative HRF/PH score (Fig. 1C). The HRF/PH score ranged 0–15 with higher score indicating increased severity.

### Data source

All data were manually extracted from the electronic health record and entered into a REDCap database [16].

### Statistical analysis

HRF/PH score was divided into 3 categories: mild (0–5), moderate (6–10), and severe (11–15), and outcomes were examined with respect to both total HRF/PH score and score category. Associations of HRF/PF scores with continuous and dichotomous outcomes were assessed using linear regression with robust standard errors and exact logistic regression, respectively. Data were summarized with absolute and relative frequencies for dichotomous outcomes and with the median and interquartile range for continuous outcomes. Fisher’s exact test was used for categorical variables with 3 or more levels. *P* < 0.05 was considered significant. Statistical analysis was performed in Stata/SE (version 17).

## Results

**Fig. 2:**
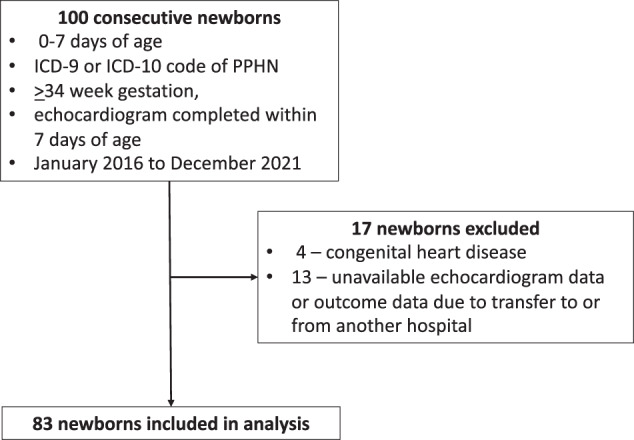
Patient flow diagram. Diagram depicts consecutive charts reviewed meeting inclusion criteria and then those that were excluded. PPHN persistent pulmonary hypertension of the newborn.

One hundred consecutive charts were reviewed, and 17 newborns were excluded – 4 due to the presence of congenital heart disease, and 13 due to missing echocardiogram or outcomes data due to transfer from or to other hospitals (Fig. 2). 83 newborns were included, of which 64% were male. The mode of delivery was cesarean section in 60% of cases. 16% of patients required suction and stimulation as their most invasive form of post-delivery resuscitation. A majority (84%) required higher levels of resuscitation, such as positive pressure ventilation (PPV) or intubation.

HRF/PH scores ranged from 2–12 with a mean of 8.5. As mentioned previously, patients were stratified into three groups to indicate their level of PPHN severity, based on their HRF/PH score. Based on the three HRF/PH score categories, 6% had mild, 77% had moderate, and 17% had severe PPHN.

Baseline demographic data were grossly similar among HRF/PH severity groups (Table 1). There were no significant differences between groups in terms of gestational age, sex assigned at birth, or delivery method. There was also no significant difference in maternal risk factors between cohorts including prolonged rupture of membranes, group B streptococcal (GBS) positive status, hypertension of pregnancy or gestational diabetes. Conditions known to cause or contribute to PPHN occurred with similar frequency among severity groups, with 36% of total cases having meconium aspiration, and 5% culture-proven sepsis. The moderate and severe PPHN patients had an increased percentage of cases with fetal distress during labor, however, it was not significant. The severe PPHN cohort had a higher number of infants with lung malformations, including CDH, congenital pulmonary airway malformation, and pulmonary hypoplasia (Table 1).

**Table 1:** Demographic characteristics by HRF/PH severity category.

	Mild (0–5) *N* = 5	Moderate (6–10) *N* = 65	Severe (11–15) *N* = 13	*p* value
Gestational age, weeks (median, IQR)	37.7 (37–38.4)	39.3 (38–40.4)	37.6 (36.9–39.9)	0.06
Sex (%)				0.21
Male	1 (20)	43 (66)	9 (69)	
Delivery method				0.22
C-section	3 (60)	42 (65)	5 (38)	
Prolonged rupture of membranes^a^	0	9/55 (16)	2/10 (20)	0.48
Fetal distress during labor	0	33 (51)	4 (31)	>0.9
GBS positive (maternal)^a^	2/2 (50)	19/54 (35)	5/9 (56)	0.59
Maternal hypertensive disease	1 (20)	14 (22)	2 (15)	0.77
Maternal diabetes	2 (40)	11 (17)	2 (15)	0.53
Post-delivery resuscitation				0.22
Suction/stimulation	2 (40)	10 (15)	1 (8)	
NIPPV	3 (60)	28 (43)	5 (38)	
Intubation	0	27 (42)	7 (54)	
Compressions required post-delivery	0	12 (18)	4 (31)	0.23
Non-cardiac birth defect	2 (40)	7 (11)	1 (8)	0.26
Genetic defect^b^	1 (20)	5 (8)	2 (15)	>0.9
Lung malformation^c^	0	2 (3)	3 (23)	**0.02**
Small for gestational age	0	7 (11)	2 (15)	0.44
Large for gestational age	0	10 (17)	4 (36)	0.10
Mechanism(s) for PPHN^d^				
Meconium aspiration	1 (20)	25 (38)	4 (31)	>0.9
Culture proven sepsis	1 (20)	2 (3)	1 (8)	>0.9
Pneumonia	0	1 (2)	0	>0.9
Hypoxic ischemic encephalopathy^e^	0	21 (32)	6 (46)	0.12
Age at echocardiogram (median, IQR)	1 (1–1)	1 (1–2)	1 (1–1)	**0.02**

^a^Denominators are shown when it differs from total column *N* due to some patients having “unknown” status.

^b^Genetic defects include all confirmed genetic abnormalities, two of which had Trisomy 21.

^c^Lung malformation includes congenital diaphragmatic hernia (*N* = 2), sequestration, congenital pulmonary airway malformation, and severe pulmonary hypoplasia.

^d^Column percentage does not equal 100% because infants may have had no identified mechanism, or multiple identified mechanisms.

^e^Hypoxic ischemic encephalopathy (HIE) includes patients confirmed to have HIE as well as though that received therapeutic cooling due to concerns and ultimately without HIE.

Bold values indicate statistical significance.

Logistic regression (Table 2) demonstrated that a higher PPHN severity category significantly correlated with combined death/ECLS, death before discharge, as well as need for iNO, IMV, HFOV, ECLS, and vasoactive infusions. 100% of cases in the severe PPHN cohort required iNO, IMV, HFOV, and vasoactive infusions, as compared to 20% of cases in the mild cohort requiring iNO and IMV, and zero cases in the mild cohort requiring HFOV or vasoactive infusions. ECLS was used in zero mild, 12% moderate, and 62% of severe PPHN cases. Finally, death before discharge was significantly increased in higher severity cohorts, with zero percent of mild, 11% of moderate, and 54% of severe PPHN cases.

**Table 2:** Outcomes by HRF/PH severity category.

	Mild (0–5) *N* = 5	Moderate (6–10) *N* = 65	Severe (11–15) *N* = 13	*p* value
Dichotomous outcomes, *N* (%)				
Death/ECLS	0	12 (18)	10 (77)	**<0.0001**
Required ECLS	0	8 (12)	8 (62)	**<0.001**
Died before discharge	0	7 (11)	7 (54)	**<0.001**
Required iNO	1 (20)	50 (77)	13 (100)	**<0.01**
Required vasoactive infusions	0	42 (65)	13 (100)	**<0.0001**
Required IMV	1 (20)	49 (75)	13 (100)	**<0.01**
Required HFOV	0	41 (84)	13 (100)	**0.03**
Ventilated week or longer^a^	0	21/42 (50)	3/6 (50)	0.72
Ventilated 2 weeks or longer^a^	0	7/42 (17)	3/6 (50)	0.13
LOS over a week^a^	4/5 (80)	53/58 (91)	6/6 (100)	0.31
Continuous outcomes^*b*^, median (IQR)				
Duration of iNO, hours^a^	8 (8–8)	86 (57–158)	120 (58–242)	0.27
Duration of IMV, days^a^	1 (1–1)	6.5 (4–11)	9 (4–16)	0.27
Total length of stay, days^a,b^	16 (13–23)	19 (11–33)	32.5 (16–76)	0.95
ICU length of stay, days^a^	16 (13–23)	19 (11–733)	32.5 (16–51)	0.93
VIS score	–	14.5 (9–27.5)	25 (15–1745)	0.17
Duration ECLS, hours^a^	–	106 (96–162)	152 (86–278)	0.69

*iNO* inhaled nitric oxide. *ECLS* extracorporeal life support. *IMV* invasive mechanical ventilation. *HFOV* high frequency oscillatory ventilation. *VIS* vasoactive-isotropic score. *IQR* interquartile range.

^a^Excludes patients that died so the denominator shown when relevant.

^b^*p* values are from linear regression comparing outcomes as continuous variables across the score categories.

Bold values indicate statistical significance.

The total mortality rate was 17%. Of the 14 patients who died, 10 of them had a progressive multiorgan failure that appeared to be incited by profound HRF and PH. Of the other 4 patients, 2 died of severe hypoxic ischemic injury with evidence of an in-utero insult and poor neurologic prognosis, 1 suffered spontaneous intestinal perforation and overwhelming sepsis in the setting of pulmonary hypoplasia, and 1 succumbed to herpes simplex virus encephalitis. Eight of the 14 patients (57%) were on ECLS prior to compassionate decannulation. The 6 patients who did not receive ECLS had known pre-existing injuries or anomalies thought to be irreversible, including severe HIE and pulmonary hypoplasia.

On linear regression analysis of infants surviving until discharge, HRF/PH score category did not correlate with duration of iNO, IMV, or ECLS, maximum VIS score, or total or ICU LOS. Outcomes are shown in Table 2. Scatter figures demonstrating the distribution of iNO, IMV, and ECLS duration, and ICU and total LOS across the total HRF/PH scores are provided in the supplement to note both some outliers as well as trends.

## Discussion

To our knowledge, our study is the first to develop and test a combined score that includes comprehensive blood gas/pulse oximetry data and echocardiogram findings to assess hypoxemia and the severity of pulmonary hypertension in PPHN. We found that our HRF/PH score category correlated with invasive ventilation, nitric oxide, high frequency ventilation, vasoactive infusions, extracorporeal life support, and death. However, the HRF/PH score category did not correlate with the duration of support or length of stay.

Classic RCTs evaluating therapy for HRF/PPHN such as iNO have used OI as entry criteria and often also as a secondary outcome [4, 5, 17] with primary outcome being death or ECLS. Authors assessing whether echocardiographic findings serve as prognostic factors have also used these primary outcomes [18–22]. Except CDH, the rates of ECLS and death have decreased for PPHN. Recruitment to trials evaluating PPHN and HRF have been difficult with many trials stopping early due to poor enrollment [23–26]. An effective strategy to enhance enrollment would be to broaden inclusion criteria to accept patients without an indwelling arterial line (to calculate OI) and use a combination of pulse oximetry and echocardiographic criteria. Our score correlates well with not only mortality and ECLS cannulation but also the need for the most intensive supportive care measures. This may be of particular interest in periods of critical shortage when it is important to gauge not only the likelihood of mortality but the need for limited resources.

We attempted to utilize commonly used echocardiographic parameters to develop the PH component of the HRF/PH score. Echocardiographic parameters to assess PPHN can be broadly classified into the assessment of (a) severity of PH or elevation of PA pressure (b) cardiac performance and (c) cardiac output. The severity of PH can be assessed by septal configuration and eccentricity index, tricuspid regurgitation (TR) jet velocity, the direction of the shunt, and RV systolic time intervals. The IVS normally bows into the right ventricle (with a O-shaped left ventricle and a crescent or D shaped right ventricle). A flat septum indicates moderate PPHN and a septum bowing into the left ventricle suggests suprasystemic pulmonary hypertension. Tricuspid regurgitation (TR) jet serves as a proxy for systolic pulmonary artery pressure (SPAP) using the modified Bernoulli equation. TR jet is an attractive measure for use in our score as it is the most direct non-invasive measure of SPAP, it is quantitative, it has been shown to correlate with poor outcomes specifically in infants with PPHN [27], and it has previously been shown to correlate to cardiac catheterization data exceptionally well in adult patients with pulmonary hypertension [27–30]. However, it cannot always be observed – it is present in approximately 60–85% of patients with PPHN and may not be visible on echocardiography in the setting of poor right ventricular contractility [5]. Additionally, its correlation with cardiac catheterization data in children under 2 appears to be poor, though such studies are extremely limited and only in specific populations such as those with chronic lung disease, and its performance is improved when used in combination with other echocardiogram findings [31]. In the current study, we used estimated RVsP in relation to systemic blood pressure to assess the severity of PPHN. TAPSE was included as the second measure of the severity of PPHN. It is a simple but highly reproducible and robust measure of right ventricular deformation that is minimally influenced by imaging artifacts, and reference values in neonates are published [32]. Additionally, Malowitz et al. found that infants with PPHN who died or needed ECLS had comparatively higher OI and lower TAPSE than those who survived without ECLS. Right ventricular dysfunction worsens the outcome of PPHN [22]. Hence the presence of right ventricular dysfunction was added as a factor.

Frequently used in other studies is bidirectional or right-to-left shunting through a PDA, which theoretically indicates pulmonary pressures near or above systemic pressures. However, shunt directionality is also influenced by intrathoracic pressure and may change minute-to-minute in response to ventilator adjustments, physical pressure, and pain or agitation. Authors that have assessed whether the directionality of shunt correlates to PPHN outcomes have found mixed results [18–20, 22], and have suggested that bidirectional shunting should not rule out severe disease. Interestingly, a more recent prospective study [33] employed serial echocardiography, showing that right-to-left ductus arteriosus shunting on second echocardiography after initial stabilization is associated with death before day 28. It is possible that the persistence of right-to-left shunting may represent a more severe disease; however, we opted not to use this measure as a single visualization is unlikely to be useful for stratification into severity categories.

Newer modalities of assessing pulmonary hypertension such as pulmonary arterial acceleration time (PAAT) and PAAT/Right ventricular ejection time (RVET) ratio are being increasingly used in the diagnosis and management of neonatal pulmonary hypertension. Left ventricular indices have also been used – however, they have largely correlated to ECLS cannulation and mortality [18, 20, 22]. As our priority was determining a range in severity of pulmonary hypertension and maintaining relative simplicity in our score, we excluded these measures.

A cardiac performance assessment was performed by assessing RV function. Including LV function as a criterion would have been beneficial as it has been linked to inadequate response to iNO and the need for ECMO. Assessing cardiac output and assessing systemic perfusion is important in PPHN. However, most traditional echocardiograms do not include LV output measurements. We did not include pulmonary arterial acceleration time (PAAT) or PAAT to right ventricular ejection time (PAAT/RVET ratio), LV dysfunction, and cardiac output at this time to maintain simplicity. We hope that the HRF/PH score can be modified in the future to include newer modalities of assessing hypoxemia, pulmonary hypertension, ventricular function and cardiac output and guide management and clinical trials in HRF/PH.

Our HRF/PH score and score categories correlate well to several outcomes that may make them useful at the bedside, as well as in research. The scoring category predicts the requirement for iNO, IMV, HFOV, vasoactive infusions, and ECLS. This could be utilized in clinical settings to facilitate timely transport to appropriate centers, particularly as ECLS remains a limited resource, and HFOV and IMV management in neonates is dependent on skilled, pediatric-specific clinicians. HRF/PH score did not correlate with ICU or total LOS; we suspect this is for multiple reasons as mentioned in the limitation section later.

A major limitation to our study is the small sample size used to test this score. Although we had a small number of patients, they were representative of HRF/PPHN patients from other studies. Similar to other studies, our patient population with moderate to severe HRF/PH showed a male predominance [34] that had associated fetal distress and required intubation for PPV during resuscitation after birth. Meconium aspiration and sepsis/pneumonia were common causes of PPHN, similar to epidemiological studies from California [35]. The precise components of the PH score may require changes to optimize its utilization in clinical practice and trials. Our criteria do not include left ventricular dysfunction that can cause pulmonary venous hypertension and worsen outcomes.

A second limitation is the wide variety of patients included in the study which may have influenced some of the results. Surgical conditions influenced length of stay [duodenal atresia repair (*n* = 2), urethral valve ablation (*n* = 1), a gastrostomy tube placement (*n* = 8), and giant omphalocele (*n* = ]1, mild PPHN but required tracheostomy with a LOS of 70 days). Future studies with larger sample sizes should attempt to control for these factors in analyzing the correlation between HRF/PH score and LOS. Our score categories also did not correlate with the duration of iNO, IMV, or ECLS. However, this may be due to a relatively smaller proportion of patients in the severe category. When assessing the duration of iNO and IMV against the total HRF/PH score as opposed to the score category, it was significant, but with few patients with an HRF/PH as the most severe end of the range. The scatter plots of score total and duration of iNO, IMV, and LOS are shown in Supplement Figures.

There are additional limitations to our study. Utilizing retrospective echocardiographic data imposed limitations with the acquisition, image quality, interpretation, and quantitative and qualitative assessment. We attempted to minimize the impact by having the cardiologist on our research team (S.S.) review all the images of the echocardiograms to standardize the results as much as possible. Furthermore, infants were scored on different days after birth depending on when an echocardiography was obtained. As pulmonary hypertension changes with time, it is possible that infants would score higher or lower if echocardiography was performed sooner or later. However, a majority of patients were scored on the first day after birth and no infants were scored later than three days from birth, meaning echocardiograms included were likely to capture PPHN at a similar acute phase without chronic structural remodeling. Additionally, the inclusion of a heterogenous sample that includes newborns with genetic conditions (i.e. Trisomy 21), HIE, and CDH, which all have different mechanisms for the pulmonary hypertension and outcome risks in themselves, may impact our findings. Future larger studies should include sub analyses that control for confounders when these complex conditions are included. Additionally, a larger study with more variation in category severity will allow for multiple regression to assess whether the severity classification provides incremental information on clinical outcomes, adjusting for co-variates. We also did not assess longer term outcomes such as neurological development, which should be considered in future studies.

In conclusion, our findings suggest that the HRF/PH score developed for the purpose of assessing PPHN offers a promising representation of the combined disease severity of hypoxemia and pulmonary hypertension during the early phrases. Validation and further refining of this score might be beneficial using large, multicenter datasets. Qualitative and implementation studies would also be helpful to evaluate the feasibility of introducing the score in a clinical practice.

### Supplementary information

Supplemental table and figures

## Data Availability

De-identified dataset will be made available by request. Requests can be submitted to the corresponding author.
