# Supplementary material for: Novel scoring tool of hypoxemic respiratory failure and pulmonary hypertension for defining severity of persistent pulmonary hypertension of newborn
Source: J Perinatol. 2023 Aug 25;43(10):1281–7. doi: 10.1038/s41372-023-01762-w (PMC10541330; doi:10.1038/s41372-023-01762-w)
Supplement: Supplementary file 1 — Supplemental table and figures [file 41372_2023_1762_MOESM1_ESM.docx]

**Supplement Table 1 – Vasoactive Isotropic Score (VIS)**

| VIS = dopamine dose (μg/kg/min) +  dobutamine dose (μg/kg/min) +  100 x epinephrine dose (μg/kg/min) +  10 x milrinone dose (μg/kg/min) +  10,000 x vasopressin dose (U/kg/min) +  100 x norepinephrine dose (μg/kg/min) |
| --- |

**Supplement Figure 1 - Scatter plots of HRF/PH Score versus Duration of iNO and mechanical ventilation**

**
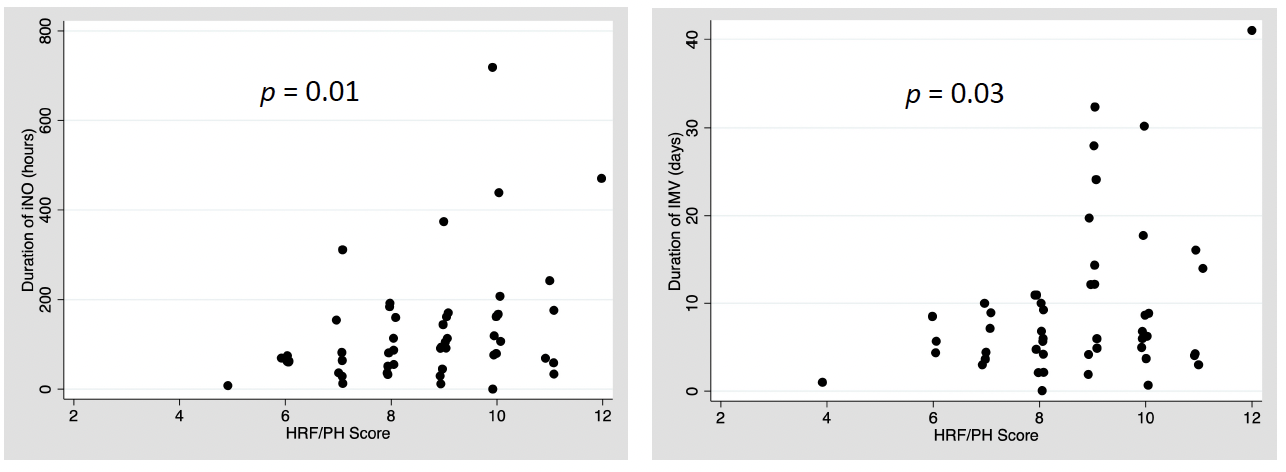
**

p values presented represent linear regression of outcome (duration iNO or mechanical ventilation) versus the HRF/PH total score as a continuous score as opposed to a categorical score. **
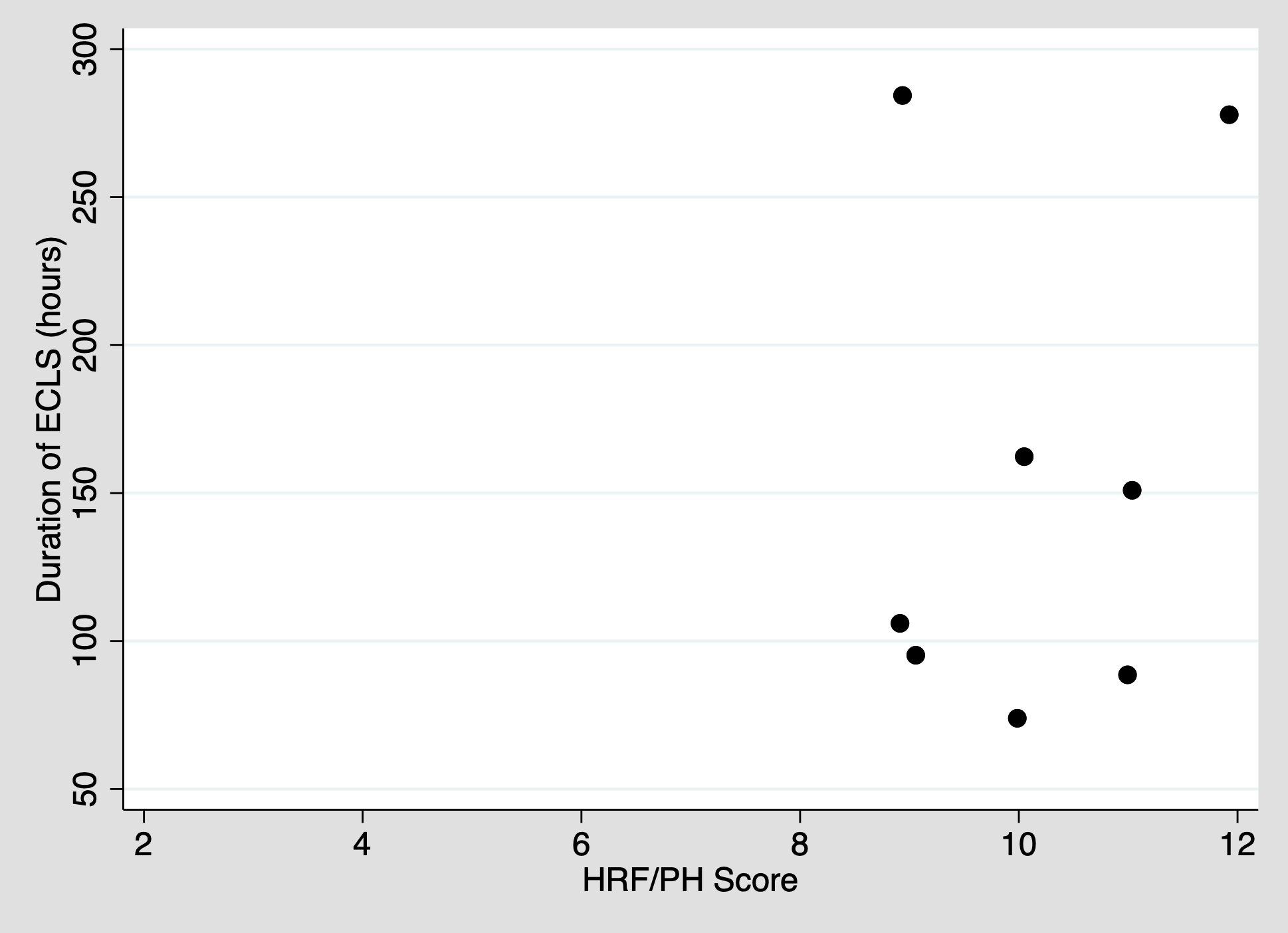
HRF/PH:** Hypoxemic Respiratory Failure/Pulmonary Hypertension Score, **iNO:** inhaled nitric oxide, **IMV**: invasive mechanical ventilation.

**Supplement Figure 2 - Scatter plots of HRF/PH Score versus Total and ICU Length of Stay**

**
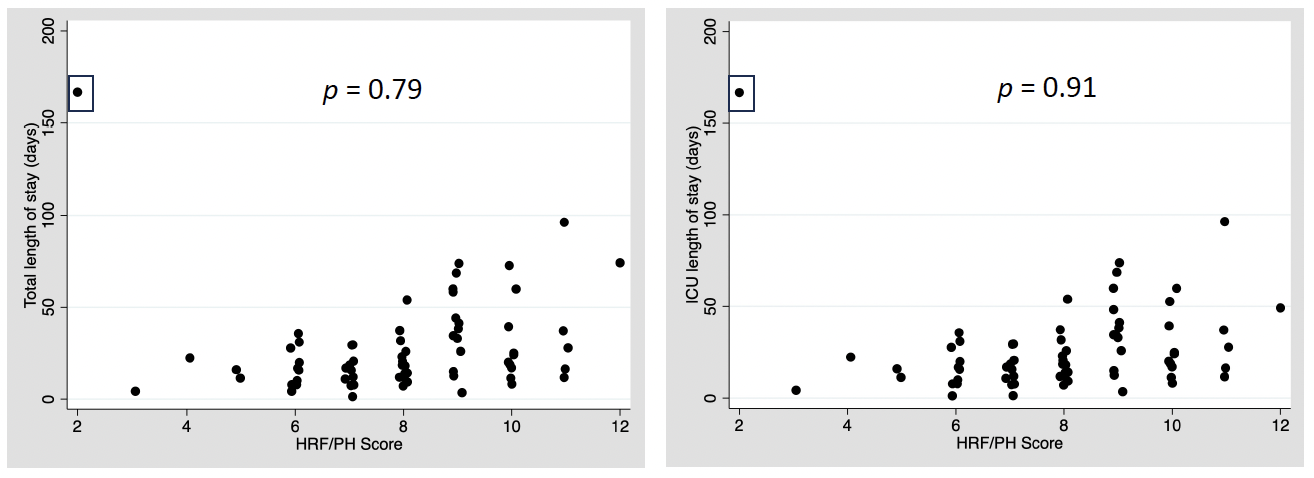
**

p values presented represent linear regression of outcome (total or ICU length of stay) versus the HRF/PH total score as a continuous score as opposed to a categorical score. Note the square is placed around an outlier patient with a low total HRF/PH (or mild) score that was noted in the manuscript. This patient with an omphalocele, scored as mild PPHN with minimal oxygen requirement, was unable to wean from positive pressure ventilation after a long period in the ICU and ultimately had a tracheostomy placed – this patient had the longest ICU and total LOS by more than 70 days. This patient appeared to have primarily hypercapnic respiratory failure, presumably in part due to inadequate ventilation from abdominal competition.**
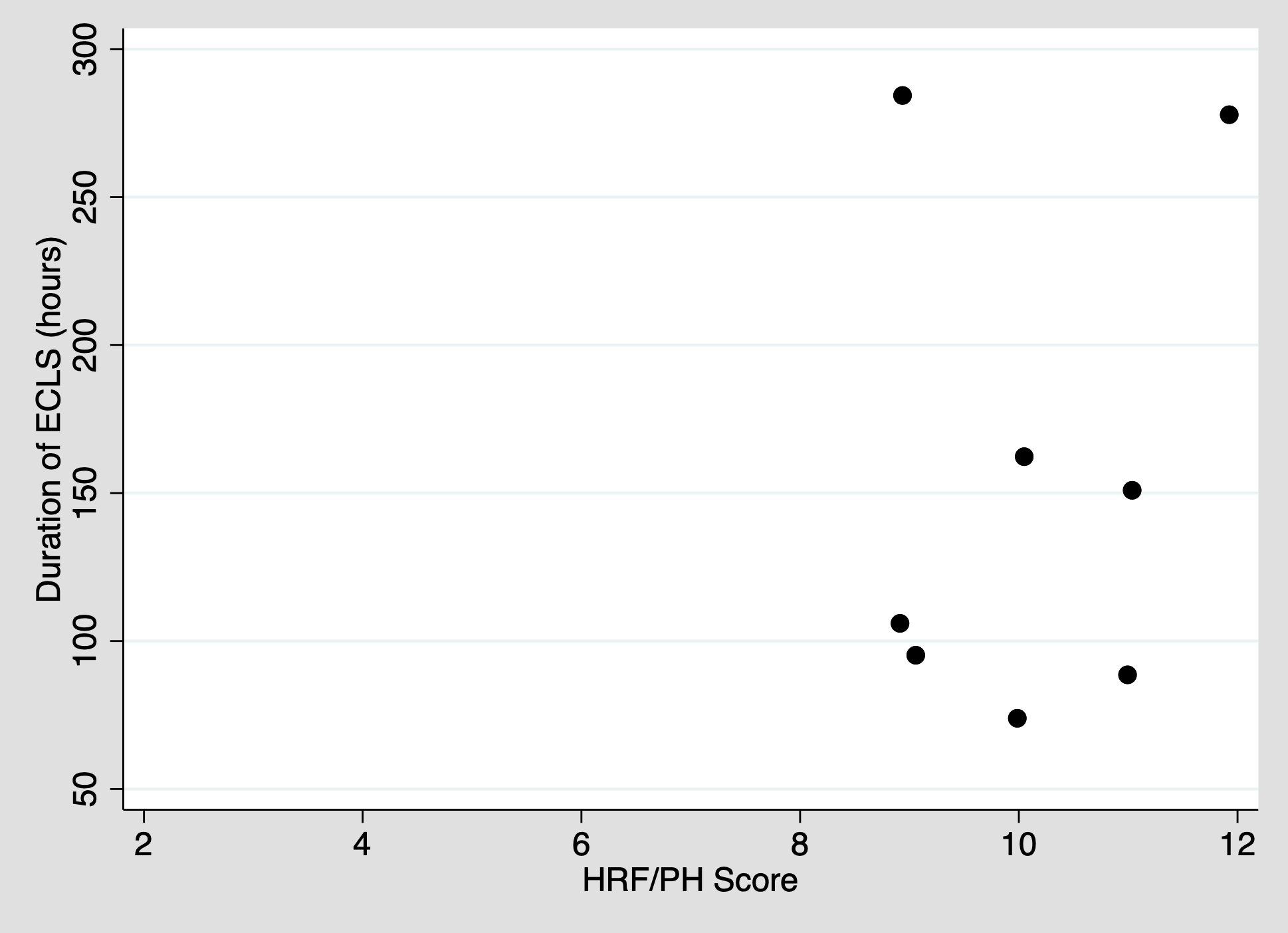
**

**HRF/PH:** Hypoxemic Respiratory Failure/Pulmonary Hypertension Score, **ICU:** Intensive Care Unit
